# Synthesis of [4-(2-Hydroxyphenyl)thiazol-2-yl]methanones as Potential Bioisosteres of Salicylidene Acylhydrazides

**DOI:** 10.3390/molecules15096019

**Published:** 2010-08-31

**Authors:** J. Mikael Hillgren, Markus K. Dahlgren, Tam M. To, Mikael Elofsson

**Affiliations:** Department of Chemistry, Umeå University, SE-90187, Umeå, Sweden

**Keywords:** type III secretion, Suzuki coupling, Grignard addition

## Abstract

A focused library of [4-(2-hydroxyphenyl)thiazol-2-yl]methanones was prepared in a four-step synthesis with the aim to obtain potent inhibitors of type III secretion in Gram-negative bacteria. The compounds are potential bioisosteres of salicylidene acylhydrazides that are a known class of type III secretion inhibitors.

## 1. Introduction

Today it is clear that resistant bacteria constitute a threat to human health and development of new antibacterial drugs is critical to extend the successful antibiotic era. Several Gram-negative pathogens including *Yersinia* spp., *Shigella* spp., *Chlamydia* spp., *Salmonella* spp., *Pseudomonas aeruginosa*, and enteropathogenic and enterohaemorrhagic *Escherichia coli* use type III secretion (T3S) of bacterial toxins to evade the human immune system and establish disease [1]. Without T3S the bacteria are essentially harmless and small molecules that inhibit this important protein export machinery therefore have the potential to be developed into novel antibacterial agents [2,3,4]. Importantly such molecules would not target bacterial growth in a non-selective manner and thereby spare the endogenous microflora. T3S inhibitors thus disarm the pathogen and allow the host immune system to clear the infection and it is expected that this mode of action will result in reduced pressure for development of resistance. This mode of action is in contrast to current antibiotics that either are bacteriostatic or bacteriocidal without distinction between pathogenic and benign endogenous bacteria.

**Figure 1:**
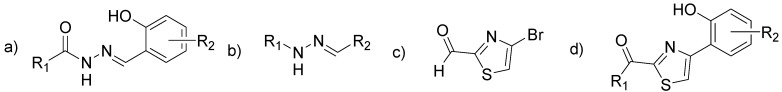
a) General structure of salicylidene acylhydrazides. b) The methylidenehydrazide linker. c) The commercially available thiazole that was identified as a possible methylidenehydrazide replacement. d) Substituted thiazoles.

Salicylidene acylhydrazides (Figure 1a) constitute a class of compounds that blocks T3S in *Y*. *pseudotuberculosis*, *Shigella flexneri*, enterohaemorrhagic *E*. *coli*, *Chlamydia* spp., and *Salmonella* spp. [5,6,7,8,9,10,11,12,13]. Their activity against multiple species indicates that T3S inhibitors potentially can be developed into novel antibacterial drugs. Activity *in vivo* has been demonstrated [11,14] and in a recent study quantitative structure-activity relationship models indicated that the pK_a_ of the salicylic aldehyde phenol part and atomic partial charges of the aromatic carbon atoms of the salicylic phenyl ring are important for T3S inhibition in *Y*. *pseudotuberculosis* [15]. Salicylidene acylhydrazides can chelate iron and it cannot be excluded that this property influences the observed T3S inhibition [9]. However, the salicylidene acylhydrazide T3S inhibitors generally suffer from some drawbacks including acid sensitivity, limited solubility and modest potency. One strategy to reduce the acid sensitivity and possibly increase the potency of the compounds is to replace the methylidenehydrazide part (Figure 1b) with another functional group. In a recent article 2-(2-aminopyrimidine)-2,2-difluoroethanols were prepared in a scaffold hopping approach to obtain possible bioisosteres [16]. This paper describes the synthesis of a library of [4-(2-hydroxyphenyl)thiazol-2-yl]methanones and the biological evaluation of this library in whole-cell *Y. pseudotuberculosis* assays.

## 2. Results and Discussion

Through commercial sources, building blocks that could mimic the methylidene hydrazide part and allow introduction of the acyl and phenol moieties were sought. A thiazole (Figure 1c) was identified as a plausible replacement of the three-atom linker of the central fragment. Interestingly, such substituted thiazoles (Figure 1d) display high structural similarity to the partially reduced thiazole skeleton in the natural product yersinabactin that is an iron chelating siderophore produced by several microorganisms, including *Yersinia* spp. [17].

**Scheme 1:**
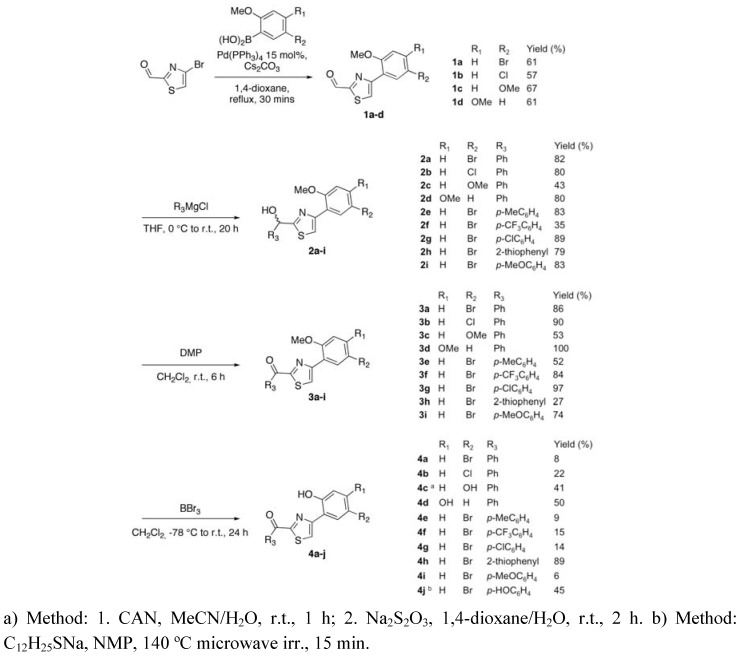
Synthesis of the thiazoles.

The thiazole compounds could be synthesized in four steps, using well-established reactions. The different building blocks and substituent patterns were selected based on structural information available for active salicylidene acylhydrazides [5,15]. Suzuki coupling under standard conditions between commercially available 4-bromothiazol-2-yl carboxaldehyde and four different boronic acids gave biaryl compounds **1a**-**d** in moderate (57-67%) yields (Scheme 1). Further functionalization of the molecular backbone was performed by addition of aryl Grignard reagents to the aldehyde moiety of **1a**-**d** to furnish alcohols **2a**-**i** (Scheme 1) in good yields (67-89%), with the exception of **2c** and **2f** that were isolated in about 40% yield. This was followed by oxidation with Dess-Martin periodinane to give ketones **3a**-**i** (Scheme 1) in good to excellent yields (52-100%). The oxidation-sensitive thiophenyl derivative **3h** could however only be isolated in 27% yield. Demethylation of **3a**-**i** with BBr_3_ furnished the salicylidene acylhydrazide mimics **4a**-**i** in low or moderate yields (typically less than 40%; Scheme 1). Ketone **3i** gave only **4i** under these conditions, while the fully deprotected analogue **4j** was obtained using an alternative procedure employing dodecylthiolate in 1-methylpyrrolidin-2-one [18]. Substrate **3c** was deprotected using cerium(III) ammonium nitrate followed by Na_2_S_2_O_3_ in an attempt to avoid supposed substrate decomposition under the harsh reaction conditions associated with the other two methods, resulting in the hydroquinone **4c** in an acceptable yield (41%). The low yields in the last step could at least in part be explained by isolation problems. Considerable loss of material was experienced following purification by both reversed and normal phase chromatography. Nevertheless, enough material of each analog was produced for biological testing. Purity of the compounds was assessed using HPLC and ^1^H-NMR and determined to be at least 90% for the intermediates (**1a**-**d**, **2a**-**i**, and **3a**-**i**) and at least 95% for the final products (**4a**-**j**). 

All target compounds **4a**-**j** and intermediates **2a-i** and **3a-i** were tested for their ability to selectively inhibit T3S using a luciferase reporter-gene assay and an assay monitoring phosphatase activity of the secreted effector protein YopH as described previously [19]. None of the compounds showed inhibitory activity in the reporter-gene assay higher than the previously set threshold of 40% at 50 μM concentration and the YopH assay confirmed lack of activity [19]. 

## 3. Experimental

### 3.1. General Chemistry

Chemicals were purchased from Sigma/Aldrich Chemical Co. and used as received unless stated otherwise. All experiments were conducted under a nitrogen atmosphere, in dried glassware, using anhydrous solvents unless stated otherwise. Microwave-heated reactions were performed at a fixed temperature using variable power in Emrys**^TM^** process vials (5 mL) using a SmithCreator**^TM^** microwave instrument from Biotage. Reagent grade dichloromethane and THF were distilled from CaH_2_ and molten potassium, respectively. Light petroleum (b.p. 40-60 ºC) is referred to as petrol.

Low-resolution mass-spectra were recorded on a Waters Micromass ZG 2000 instrument with an electro spray ion source (ES+ and ES-) and using an XTerra® MS C_18_ 5 μm particle size, 4.6 × 50 mm column and a water/acetonitrile/0.2% formic acid eluent system. High-resolution mass-spectra were recorded on a Waters Micromass GCT instrument with an electron impact ion source (EI+), direct inlet (20-400 deg.), or Bruker micrOTOF II with an electro-spray ion source (ES+). Preparative reversed-phase HPLC was performed on a Beckman System Gold HPLC with a Supelco Discovery BIO Wide Pore C_18_ column, using a water/acetonitrile/0.1% trifluoroacetic acid eluent system with a flow rate of 11 mL min^-1^ and detection at 214 nm. ^1^H-NMR and ^13^C-NMR spectra were recorded at 298 K on Bruker DRX-360 (^1^H: 360 MHz; ^13^C: 90 MHz), DRX-400 (^1^H: 400 MHz; ^13^C: 100 MHz) or DRX-500 (^1^H: 500 MHz; ^13^C: 125 MHz) spectrometers, with chemical shift values being reported in ppm relative to residual CHCl_3_ (7.26 (δ_H_) and 77.16 (δ_C_) ppm), CD_3_OH (3.31 (δ_H_) and 49.00 (δ_C_) ppm) or acetone-*d*_5_ (2.05 (δ_H_) and 29.84 (δ_C_) ppm) as internal standard. Peak assignments could be established from complementary HMBC, HSQC and COSY experiments. Infrared spectra were recorded using a customized Perkin Elmer Spectrum BX FT-IR/ATR spectrophotometer. Melting points were measured using a Büchi/Dr. Tottoli melting point determination apparatus and are uncorrected.

### 3.2. Synthesis

#### 3.2.1. General procedure for Suzuki coupling

4-Bromothiazole-2-carboxaldehyde (538 mg, 2.80 mmol), tetrakis(triphenylphosphine)palladium(0) (485 mg, 0.410 mmol) and caesium carbonate (2.01 g, 6.17 mmol) were dissolved in 1,4-dioxane (20 mL) and the resulting suspension stirred at r.t. for 5 min. The relevant boronic acid (5.59 mmol) was added and the resulting mixture refluxed for 30 min. After cooling to ambient temperature brine (10 mL) was added to the mixture, the layers were separated, and the aqueous layer was extracted with ethyl acetate (3 × 10 mL). The combined organic layers were dried (Na_2_SO_4_) and concentrated, and the residue was purified by silica gel chromatography (1:9 ethyl acetate-heptanes) to give the aldehydes **1a-d**.

*4-(5-Bromo-2-methoxyphenyl)thiazole-2-carboxaldehyde* (**1a**). Obtained on a 2.80 mmol scale as a pale yellow solid (511 mg, 61%). An analytical sample was recrystallized from chloroform to give pale yellow needles, m.p. 122.5-126.0 ºC (chloroform); R_f_ 0.35 (1:1 diethyl ether-petrol); IR ν_max_/cm^-1^ 3329, 3155, 2945, 2844, 1681, 1670; ^1^H-NMR δ_H_ (400 MHz, CDCl_3_) 10.06 (s, 1H), 8.43 (d, *J* = 2.5 Hz, 1H), 8.35 (d, *J* = 1.2 Hz, 1H), 7.45 (dd, *J* = 8.8, 2.5 Hz, 1H), 6.91 (d, *J* = 8.8 Hz, 1H), 3.96 (s, 3H); ^13^C-NMR δ_C_ (100 MHz, CDCl_3_) 184.1, 164.1, 156.1, 152.9, 132.7, 132.5, 125.2, 124.0, 113.6, 113.1, 56.0; *m/z* LRMS (ES+) 300.29 (100%, [M(^81^Br)+H]^+^), 298.28 (96, [M(^79^Br)+H]^+^), 155.16 (85), 149.17 (85).

*4-(5-Chloro-2-methoxyphenyl)thiazole-2-carboxaldehyde* (**1b**). Obtained on a 4.00 mmol scale as a pale yellow solid (579 mg, 57%). An analytical sample was recrystallized from chloroform to give pale yellow needles, m.p. 125.0-127.0 ºC (chloroform); R_f_ 0.35 (1:3 ethyl acetate-petrol); IR ν_max_/cm^-1^ 3327, 3158, 2949, 2846, 1680, 1670; ^1^H-NMR δ_H_ (400 MHz, CDCl_3_) 10.06 (d, *J* = 1.3 Hz, 1H), 8.36 (d, *J* = 1.3 Hz, 1H), 8.29 (d, *J* = 2.7 Hz, 1H), 7.31 (dd, *J* = 8.8, 2.7 Hz, 1H), 6.95 (d, *J* = 8.8 Hz, 1H), 3.96 (s, 3H); ^13^C-NMR δ_C_ (100 MHz, CDCl_3_) 184.1, 164.1, 155.7, 153.1, 129.8, 129.5, 126.4, 125.2, 123.5, 112.7, 56.0; *m/z* LRMS (ES+) 256.31 (39%, [M(^37^Cl)+H]^+^), 254.30 (100, [M(^35^Cl)+H]^+^), 239.25 (41).

*4-(2,5-Dimethoxyphenyl)thiazole-2-carboxaldehyde* (**1c**). Obtained on a 0.99 mmol scale as a pale yellow solid (165 mg, 67%). An analytical sample was recrystallized from chloroform to give pale yellow needles, m.p. 88.5-89.0 ºC (chloroform); R_f_ 0.33 (1:3 ethyl acetate-petrol); IR ν_max_/cm^-1^ 3152, 2994, 2832, 1676; ^1^H-NMR δ_H_ (400 MHz, CDCl_3_) 10.05 (d, *J* = 1.3 Hz, 1H), 8.36 (d, *J* = 1.3 Hz, 1H), 7.87 (d, *J* = 2.9 Hz, 1H), 6.95 (d, *J* = 8.9 Hz, 1H), 6.91 (dd, *J* = 8.9, 2.9 Hz, 1H), 3.90 (s, 3H), 3.86 (s, 3H); ^13^C-NMR δ_C_ (100 MHz, CDCl_3_) 184.2, 163.8, 154.2, 153.9, 151.4, 124.7, 122.7, 115.7, 114.8, 112.7, 56.1, 56.0; *m/z* LRMS (ES+) 250.27 (76%, [M+H]^+^), 235.22 (100), 239.25 (29).

*4-(2,4-Dimethoxyphenyl)thiazole-2-carboxaldehyde* (**1d**). Obtained on a 4.00 mmol scale as a pale yellow solid (605 mg, 61%). An analytical sample was recrystallized from chloroform to give pale yellow needles, m.p. 110.5-116.0 ºC (chloroform); R_f_ 0.30 (1:3 ethyl acetate-petrol); IR ν_max_/cm^-1^ 3142, 2836, 1669, 1607, 1575; ^1^H-NMR δ_H_ (400 MHz, CDCl_3_) 10.05 (d, *J* = 1.3 Hz, 1H), 8.21 (d, *J* = 8.7 Hz, 1H), 8.19 (d, *J* = 1.3 Hz, 1H), 6.63 (dd, *J* = 8.7, 2.4 Hz, 1H), 6.57 (d, *J* = 2.4 Hz, 1H), 3.93 (s, 3H), 3.86 (s, 3H); ^13^C-NMR δ_C_ (100 MHz, CDCl_3_) 184.2, 163.7, 161.3, 158.2, 154.6, 131.0, 122.6, 115.5, 105.2, 98.9, 55.63, 55.59; *m/z* LRMS (ES+) 279.41 (28%), 264.38 (67), 250.34 (100, [M+H]^+^).

#### 3.2.2. General procedure for Grignard addition

The appropriate aldehyde (1.00 mmol) was dissolved in THF (20 mL) and cooled to 0 ºC. Arylmagnesium bromide (1-2 M solution in THF; 1.50 mmol) was added dropwise over the course of 5 min. The resulting mixture was stirred for 3 h, followed by further addition of arylmagnesium bromide (1-2 M solution in THF; 0.500 mmol). After stirring for another 3 h, the mixture was allowed to warm to r.t. over the course of 14 h. The reaction was quenched by addition of water (20 mL) and concentrated acetic acid (until pH ≈ 5), the resulting mixture stirred for 10 min, then extracted with ethyl acetate (3 × 20 mL). The combined organic layers were dried (Na_2_SO_4_) and concentrated and the residue was purified by silica gel chromatography (1:9 ethyl acetate-heptanes) to give the alcohols **2a-i**.

*[4-(5-Bromo-2-methoxyphenyl)thiazol-2-yl](phenyl)methanol* (**2a**). Obtained on a 1.00 mmol scale as a colourless wax (310 mg, 82%); R_f_ 0.12 (1:3 ethyl acetate-heptanes); IR ν_max_/cm^-1^ 3409, 1592, 1573; ^1^H-NMR δ_H_ (400 MHz, CDCl_3_) 8.38 (d, *J* = 2.6 Hz, 1H), 7.91 (s, 1H), 7.52 (dd, *J* = 8.3, 1.6 Hz, 2H), 7.42-7.32 (m, 4H), 6.85 (d, 8.8 Hz, 1H), 6.09 (s, 1H), 3.91 (s, 3H); ^13^C-NMR δ_C_ (100 MHz, CDCl_3_) 172.1, 155.9, 149.1, 141.4, 132.6, 131.5, 128.9, 128.7, 126.8, 124.7, 119.0, 113.5, 113.0, 74.0, 55.9; *m/z* LRMS (ES+) 378.37 (10%, [M(^81^Br)+H]^+^), 376.36 (10, [M(^79^Br)+H]^+^), 360.36 (100, [M(^81^Br)–OH]^+^), 358.35 (95, [M(^79^Br)–OH]^+^).

*[4-(5-Chloro-2-methoxyphenyl)thiazol-2-yl](phenyl)methanol* (**2b**). Obtained on a 1.93 mmol scale as a colourless wax (511 mg, 80%); R_f_ 0.19 (1:3 ethyl acetate-heptanes); IR ν_max_/cm^-1^ 3338, 1494, 1476, 1452; ^1^H-NMR δ_H_ (400 MHz, CDCl_3_) 8.23 (d, *J* = 2.7 Hz, 1H), 7.91 (s, 1H), 7.52 (dtd, *J* = 6.9, 1.4, 0.5 Hz, 2H), 7.39 (ddt, *J* = 7.2, 6.9, 1.5 Hz, 2H), 7.34 (tt, *J* = 7.2, 1.4 Hz, 1H), 7.23 (dd, *J* = 8.8, 2.7 Hz, 1H), 6.88 (d, *J* = 8.8 Hz, 1H), 6.08 (s, 1H), 3.90 (s, 3H); ^13^C-NMR δ_C_ (100 MHz, CDCl_3_) 172.1, 155.4, 149.2, 141.4, 129.7, 128.8, 128.6, 128.5, 126.8, 126.0, 124.4, 119.0, 112.4, 74.0, 55.9; *m/z* LRMS (ES+) 332.34 (10%, [M(^35^Cl)+H]^+^), 316.34 (52, [M(^37^Cl)–OH]^+^), 314.32 (100, [M(^35^Cl)–OH]^+^).

*[4-(2,5-Dimethoxyphenyl)thiazol-2-yl](phenyl)methanol* (**2c**). Obtained on a 1.54 mmol scale as a pale yellow wax (217 mg, 43%); R_f_ 0.10 (1:3 ethyl acetate-heptanes); IR ν_max_/cm^-1^ 3396, 2919, 2849, 2831, 1502, 1452; ^1^H-NMR δ_H_ (400 MHz, CDCl_3_) 7.93 (s, 1H), 7.85 (d, *J* = 3.0 Hz, 1H), 7.52 (d, *J* = 7.1 Hz, 2H), 7.38 (tt, *J* = 7.1, 1.7 Hz, 2H), 7.33 (tt, *J* = 7.2, 1.4 Hz, 1H), 6.92 (d, *J* = 8.9 Hz, 1H), 6.85 (dd, *J* = 8.9, 3.0 Hz, 1H), 6.10 (s, 1H), 3.88 (s, 3H), 3.84 (s, 3H); ^13^C-NMR δ_C_ (100 MHz, CDCl_3_) 171.9, 153.8, 151.3, 150.2, 141.5, 128.8, 128.6, 126.8, 123.5, 118.4, 115.3, 114.5, 112.6, 73.9, 56.1, 56.0; *m/z* LRMS (ES+) 328.33 (21%, [M+H]^+^), 311.30 (50), 310.33 (100, [M–OH]^+^).

*[4-(2,4-Dimethoxyphenyl)thiazol-2-yl](phenyl)methanol* (**2d**). Obtained on a 2.00 mmol scale as a colourless solid (523 mg, 80%). An analytical sample was recrystallized from chloroform to give colourless plates, m.p. 132.5-133.5 ºC (chloroform); R_f_ 0.19 (1:3 ethyl acetate-heptanes); IR ν_max_/cm^-1^ 3149, 1612, 1582; ^1^H-NMR δ_H_ (400 MHz, CDCl_3_) 8.17 (d, *J* = 8.6 Hz, 1H), 7.74 (s, 1H), 7.52 (dd, *J* = 8.3, 1.2 Hz, 2H), 7.41-7.31 (m, 3H), 6.59 (dd, *J* = 8.6, 2.4 Hz, 1H), 6.55 (d, *J* = 2.4 Hz, 1H), 6.08 (s, 1H), 3.91 (s, 3H), 3.85 (s, 3H); ^13^C-NMR δ_C_ (100 MHz, CDCl_3_) 171.5, 160.6, 158.0, 150.4, 141.6, 131.0, 128.8, 128.6, 126.9, 116.0, 104.9, 98.9, 74.0, 55.6 (2 signals); *m/z* LRMS (ES+) 328.31 (24%, [M+H]^+^), 311.30 (48), 310.33 (100, [M–OH]^+^).

*[4-(5-Bromo-2-methoxyphenyl)thiazol-2-yl](4-methylphenyl)methanol* (**2e**). Obtained on a 1.00 mmol scale as a pale yellow wax (273 mg, 67%); R_f_ 0.32 (1:3 ethyl acetate-heptanes); IR ν_max_/cm^-1^ 3329, 2921, 2849, 1490, 1243, 1019; ^1^H-NMR δ_H_ (360 MHz, CDCl_3_) 8.37 (d, *J* = 2.6 Hz, 2H), 7.90 (s, 1H), 7.41 (d, *J* = 8.0 Hz, 2H), 7.39 (dd, *J* = 8.9, 2.6 Hz, 1H), 7.20 (d, *J* = 7.9 Hz, 1H), 6.85 (d, *J* = 8.9 Hz, 1H), 6.07 (s, 1H), 3.92 (s, 3H), 2.36 (s, 3H); ^13^C-NMR δ_C_ (90 MHz, CDCl_3_) 172.9, 156.3, 149.2, 138.84, 138.76, 133.0, 131.9, 129.9, 127.1, 124.9, 119.3, 113.8, 113.3, 74.0, 56.2, 21.7; *m/z* LRMS (ES+) 374.28 (100%, [M(^81^Br)-OH]^+^), 372.27 (92, [M(^79^Br)-OH]^+^).

*[4-(5-Bromo-2-methoxyphenyl)-thiazol-2-yl](4-trifluoromethylphenyl)methanol* (**2f**). Obtained on a 1.00 mmol scale as a pale yellow oil (155 mg, 35%); R_f_ 0.31 (1:3 ethyl acetate-heptanes); IR ν_max_/cm^-1^ 3146, 2937, 2840, 1492, 1322, 1247; ^1^H-NMR δ_H_ (360 MHz, CDCl_3_) 8.33 (d, *J* = 2.6 Hz, 1H), 7.91 (s, 1H), 7.67-7.61 (m, 4H), 7.38 (dd, *J* = 8.8, 2.6 Hz, 1H), 6.84 (d, *J* = 8.8 Hz, 1H), 6.16 (s, 1H), 3.90 (s, 3H); ^13^C-NMR δ_C_ (90 MHz, CDCl_3_) 171.3, 155.9, 149.3, 145.1, 132.6, 131.7, 130.7 (q, ^2^*J*_CF_ = 32.5 Hz), 127.0, 125.8 (q, ^3^*J*_CF_ = 3.4 Hz), 124.5, 124.1 (q, ^1^*J*_CF_ = 272.4 Hz), 119.2, 113.4, 113.0, 73.2, 55.8; *m/z* LRMS (ES+) 428.19 (100%, [M(^81^Br)-OH]^+^), 426.24 (96, [M(^79^Br)-OH]^+^).

*[4-(5-Bromo-2-methoxyphenyl)-thiazol-2-yl](4-chlorophenyl)methanol* (**2g**). Obtained on a 1.00 mmol scale as a colourless oil (365 mg, 89%); R_f_ 0.49 (1:3 ethyl acetate-heptanes); IR ν_max_/cm^-1^ 3143, 2935, 2837, 1489, 1245; ^1^H-NMR δ_H_ (400 MHz, CDCl_3_) 8.34 (d, *J* = 2.6 Hz, 1H), 7.91 (s, 1H), 7.46-7.33 (m, 5H), 6.85 (d, *J* = 8.8 Hz, 1H), 6.07 (s, 1H), 3.91 (s, 3H); ^13^C-NMR δ_C_ (100 MHz, CDCl_3_) 171.5, 155.8, 149.1, 139.7, 134.4, 132.5, 131.5, 128.9, 128.1, 124.5, 119.0, 113.3, 112.9, 73.1, 55.8; *m/z* LRMS (ES+) 396.26 (30%, [M(^37^Cl^81^Br)-OH]^+^), 394.25 (100, [M(^35^Cl^81^Br)-OH]^+^,[M(^37^Cl^79^Br)-OH]^+^), 392.30 (72, [M(^35^Cl^79^Br)-OH]^+^).

*[4-(5-Bromo-2-methoxyphenyl)thiazol-2-yl](thiophen-2-yl)methanol* (**2h**). Obtained on a 1.00 mmol scale as a yellow wax (300 mg, 79%); R_f_ 0.36 (1:3 ethyl acetate-heptanes); IR ν_max_/cm^-1^ 3232, 3157, 2834, 2360, 1498, 1473, 1458, 1436; ^1^H-NMR δ_H_ (400 MHz, CDCl_3_) 8.37 (d, *J* = 2.6 Hz, 1H), 7.94 (s, 1H), 7.37 (dd, *J* = 8.8, 2.6 Hz, 1H), 7.32 (dd, *J* = 5.1, 1.2 Hz, 1H), 7.15 (ddd, *J* = 3.5, 1.2, 0.8 Hz, 1H), 7.01 (dd, *J* = 5.1, 3.5 Hz, 1H), 6.85 (d, *J* = 8.8 Hz, 1H), 6.35 (d, *J* = 0.8 Hz, 1H), 3.92 (s, 3H); ^13^C-NMRδ_C_ (100 MHz, CDCl_3_) 170.9, 155.9, 149.1, 144.8, 132.6, 131.5, 126.9, 126.3, 125.8, 124.6, 119.0, 113.4, 112.9, 70.0, 55.8; *m/z* LRMS (ES+) 366.28 (100%, [M(^81^Br)-OH]^+^), 364.27 (91, [M(^79^Br)-OH]^+^).

*[4-(5-Bromo-2-methoxyphenyl)thiazol-2-yl](4-methoxyphenyl)methanol* (**2i**). Obtained on a 1.00 mmol scale as a colourless oil (337 mg, 83%); R_f_ 0.21 (1:3 ethyl acetate-heptanes); IR ν_max_/cm^-1^ 3371, 3143, 2933, 2836, 1610, 1589, 1509, 1242; ^1^H-NMR δ_H_ (400 MHz, CDCl_3_) 8.36 (d, *J* = 2.6 Hz, 1H), 7.89 (s, 1H), 7.41 (d, *J* = 8.6 Hz, 2H), 7.36 (dd, *J* = 8.8, 2.6 Hz, 1H), 6.90 (d, *J* = 8.6 Hz, 2H), 6.83 (d, *J* = 8.8 Hz, 1H), 6.02 (s, 1H), 3.89 (s, 3H), 3.80 (s, 3H); ^13^C-NMR δ_C_ (100 MHz, CDCl_3_) 173.0, 160.1, 156.2, 149.4, 134.1, 132.9, 131.7, 128.5, 125.5, 119.3, 114.5, 113.7, 113.3, 73.9, 56.2, 55.7; *m/z* LRMS (ES+) 390.29 (100%, [M(^81^Br)-OH]^+^), 388.29 (91, [M(^79^Br)-OH]^+^).

#### 3.2.3. General procedure for oxidation of secondary alcohols with Dess-Martin periodinane

The relevant alcohol (0.550 mmol) was dissolved in dichloromethane (2.5 mL) and Dess-Martin periodinane (15% solution in dichloromethane; 2.39 mL, 1.10 mmol) was added in one portion. The resulting solution was stirred at r.t. for 6 h, followed by addition of 1 M aqueous NaOH (4 mL). After stirring the resulting biphasic mixture for 5 min., diethyl ether was added (30 mL), the phases separated and the aqueous layer extracted with diethyl ether (2 × 10 mL). The combined organic layers were dried (Na_2_SO_4_) and concentrated, and the resulting crude residue filtered through a short plug of silica gel (eluting with 1:19 ethyl acetate-heptanes) to give the ketones **3a-i**.

*[4-(5-Bromo-2-methoxyphenyl)thiazol-2-yl](phenyl)methanone* (**3a**). Obtained on a 0.550 mmol scale as a pale yellow solid (202 mg, 86%). An analytical sample was recrystallized from chloroform to give yellow needles, m.p. 131.0-133.0 ºC (chloroform); R_f_ 0.34 (1:3 ethyl acetate-heptanes); IR ν_max_/cm^-1^ 3144, 3062, 3001, 2839, 1630, 1592, 1574; ^1^H-NMR δ_H_ (360 MHz, CDCl_3_) 8.57 (dtd, *J* = 7.6, 1.4, 0.6 Hz, 2H), 8.37 (d, *J* = 2.6 Hz, 1H), 8.32 (s, 1H), 7.67 (tt, *J* = 7.4, 1.4 Hz, 1H), 7.57 (t, *J* = 7.6 Hz, 2H), 7.43 (dd, *J* = 8.8, 2.6 Hz, 1H), 6.89 (d, *J* = 8.8 Hz, 1H), 3.96 (s, 3H); ^13^C-NMR δ_C_ (90 MHz, CDCl_3_) 183.9, 165.7, 156.1, 152.0, 135.2, 133.7, 132.6, 132.0, 131.3, 128.5, 125.4, 124.4, 113.5, 113.1, 55.9; *m/z* LRMS (ES+) 376.29 (100%, [M^81^Br+H]^+^), 374.28 (94, [M^79^Br+H]^+^).

*[4-(5-Chloro-2-methoxyphenyl)thiazol-2-yl](phenyl)methanone* (**3b**). Obtained on a 1.36 mmol scale as a pale yellow solid (405 mg, 90%). An analytical sample was recrystallized from dichloromethane to give yellow needles, m.p. 132.0-133.0 ºC (dichloromethane); R_f_ 0.30 (1:3 ethyl acetate-heptanes); IR ν_max_/cm^-1^ 3144, 3067, 3006, 2838, 1630, 1593, 1574; ^1^H-NMR δ_H_ (360 MHz, CDCl_3_) 8.57 (dd, *J* = 7.2, 1.3 Hz, 2H), 8.32 (s, 1H), 8.23 (d, *J* = 2.7 Hz, 1H), 7.67 (tt, *J* = 7.4, 1.3 Hz, 1H), 7.57 (tt, *J* = 7.3, 1.3 Hz, 2H), 7.28 (dd, *J* = 8.8, 2.7 Hz, 1H), 6.92 (d, *J* = 8.8 Hz, 1H), 3.95 (s, 3H); ^13^C-NMR δ_C_ (90 MHz, CDCl_3_) 184.0, 165.7, 155.7, 152.1, 135.2, 133.7, 131.3, 129.8, 129.1, 128.5, 126.1, 125.4, 124.0, 112.6, 55.9; *m/z* LRMS (ES+) 332.26 (38%, [M^37^Cl+H]^+^), 330.26 (100, [M^35^Cl+H]^+^).

*[4-(2,5-Dimethoxyphenyl)thiazol-2-yl](phenyl)methanone* (**3c**). Obtained on a 0.580 mmol scale as a pale yellow solid (101 mg, 53%). An analytical sample was recrystallized from chloroform to give yellow needles, m.p. 107.5-109.5 ºC (chloroform); R_f_ 0.30 (1:3 ethyl acetate-heptanes); IR ν_max_/cm^-1^ 3122, 2995, 2827, 1638; ^1^H-NMR δ_H_ (360 MHz, CDCl_3_) 8.61 (dd, *J* = 7.3, 1.2 Hz, 2H), 8.34 (s, 1H), 7.88 (d, *J* = 3.0 Hz, 1H), 7.64 (t, *J* = 7.4 Hz, 1H), 7.54 (t, *J* = 7.6 Hz, 2H), 6.94 (d, *J* = 9.0 Hz, 1H), 6.88 (dd, *J* = 9.0, 3.0 Hz, 1H), 3.91 (s, 3H), 3.84 (s, 3H); ^13^C-NMR δ_C_ (90 MHz, CDCl_3_) 171.9, 153.8, 151.3, 150.2, 141.5, 128.8, 128.6, 126.8, 123.5, 118.4, 115.3, 114.5, 112.6, 73.9, 56.1, 56.0; *m/z* LRMS (ES+) 326.32 (100%, [M+H]^+^).

*[4-(2,4-Dimethoxyphenyl)thiazol-2-yl](phenyl)methanone* (**3d**). Obtained on a 1.12 mmol scale as a pale yellow solid (363 mg, 100%). An analytical sample was recrystallized from chloroform to give yellow needles, m.p. 106.0-107.5 ºC (chloroform); R_f_ 0.30 (1:3 ethyl acetate-heptanes); IR ν_max_/cm^-1^ 3141, 3071, 1643, 1611, 1597, 1578; ^1^H-NMR δ_H_ (360 MHz, CDCl_3_) 8.61 (dd, *J* = 8.3, 1.3 Hz, 2H), 8.22 (d, *J* = 8.6 Hz, 1H), 8.17 (s, 1H), 7.63 (tt, *J* = 7.4, 1.3 Hz, 1H), 7.54 (t, *J* = 7.6 Hz, 2H), 6.63 (dd, *J* = 8.6, 2.4 Hz, 1H), 6.56 (d, *J* = 2.4 Hz, 1H), 3.93 (s, 3H), 3.85 (s, 3H); ^13^C-NMR δ_C_ (90 MHz, CDCl_3_) 184.4, 165.6, 161.4, 158.6, 154.1, 135.9, 133.9, 131.8, 131.4, 128.8, 123.0, 116.4, 105.5, 99.1, 55.9 (2 signals); *m/z* LRMS (ES+) 326.29 (100%, [M+H]^+^).

*[4-(5-Bromo-2-methoxyphenyl)-thiazol-2-yl](4-methylphenyl)methanone* (**3e**). Obtained on a 0.64 mmol scale as a yellow solid (130 mg, 52%). An analytical sample was recrystallized from chloroform to give yellow needles, m.p. 136.0-138.0 ºC (chloroform); R_f_ 0.42 (1:3 ethyl acetate-heptanes); IR ν_max_/cm^-1^ 3145, 2838, 1626, 1603, 1567; ^1^H-NMR δ_H_ (400 MHz, CDCl_3_) 8.50 (d, *J* = 8.2 Hz, 2H), 8.38 (d, *J* = 2.3 Hz, 1H), 8.30 (s, 1H), 7.43 (dd, *J* = 8.8, 2.3 Hz, 1H), 7.37 (d, *J* = 8.2 Hz, 2H), 6.89 (d, *J* = 8.8 Hz, 1H), 3.96 (s, 3H), 2.48 (s, 3H); ^13^C-NMR δ_C_ (100 MHz, CDCl_3_) 183.6, 166.1, 156.2, 151.9, 144.8, 132.70, 132.66, 132.0, 131.5, 129.3, 125.2, 124.6, 113.5, 113.1, 55.9, 22.0; *m/z* LRMS (ES+) 390.28 (100%, [M(^81^Br)+H]^+^), 388.27 (92, [M(^79^Br)+H]^+^).

*[4-(5-Bromo-2-methoxyphenyl)thiazol-2-yl](4-trifluoromethylphenyl)methanone* (**3f**). Obtained on a 0.64 mmol scale as a yellow solid (240 mg, 84%). An analytical sample was recrystallized from chloroform to give yellow needles, m.p. 144.5-145.5 ºC (chloroform); R_f_ 0.39 (1:3 ethyl acetate-heptanes); IR ν_max_/cm^-1^ 3138, 3089, 3016, 2946, 2842, 1645; ^1^H-NMR δ_H_ (360 MHz, CDCl_3_) 8.66 (d, *J* = 8.1 Hz, 2H), 8.36 (s, 1H), 8.31 (d, *J* = 2.6 Hz, 1H), 7.83 (d, *J* = 8.1 Hz, 2H), 7.45 (dd, *J* = 8.8, 2.6 Hz, 1H), 6.90 (d, *J* = 8.8 Hz, 1H), 3.97 (s, 3H); ^13^C-NMR δ_C_ (90 MHz, CDCl_3_) 183.3, 165.3, 156.5, 152.7, 138.4, 135.1 (q, ^2^*J*_CF_ = 32.5 Hz), 132.9, 132.7, 131.9, 126.3, 125.8 (q, ^3^*J*_CF_ = 4.1 Hz), 124.5, 124.1 (q, ^1^*J*_CF_ = 273.4 Hz), 113.9, 113.5, 56.3; *m/z* LRMS (ES+) 444.19 (100%, [M(^81^Br)+H]^+^), 442.18 (92, [M(^79^Br)+H]^+^).

*[4-(5-Bromo-2-methoxyphenyl)thiazol-2-yl](4-chlorophenyl)methanone* (**3g**). Obtained on a 0.75 mmol scale as a yellow solid (300 mg, 97%). An analytical sample was recrystallized from chloroform to give yellow needles, m.p. 150.5-153.0 ºC (chloroform); R_f_ 0.46 (1:3 ethyl acetate-heptanes); IR ν_max_/cm^-1^ 3147, 3081, 2836, 1635, 1584; ^1^H-NMR δ_H_ (360 MHz, CDCl_3_) 8.54 (d, *J* = 8.7 Hz, 2H), 8.32 (s, 1H), 8.32 (d, *J* = 2.6 Hz, 1H), 7.54 (d, *J* = 8.7 Hz, 2H), 7.43 (dd, *J* = 8.8, 2.6 Hz, 1H), 6.89 (d, *J* = 8.8 Hz, 1H), 3.96 (s, 3H); ^13^C-NMR δ_C_ (100 MHz, CDCl_3_) 183.0, 165.7, 156.5, 152.5, 140.8, 133.8, 133.0, 132.9, 132.5, 129.2, 126.1, 124.6, 113.8, 113.4, 56.3; *m/z* LRMS (ES+) 412.13 (30%, [M(^37^Cl^81^Br)+H]^+^), 410.18 (100, [M(^35^Cl^81^Br)+H]^+^, [M(^37^Cl^79^Br)+H]^+^), 408.17 (75, [M(^35^Cl^79^Br)+H]^+^).

*[4-(5-Bromo-2-methoxyphenyl)thiazol-2-yl](thiophen-2-yl)methanone* (**3h**). Obtained on a 0.72 mmol scale as a yellow solid (75 mg, 27%). An analytical sample was recrystallized from chloroform to give yellowish brown needles, m.p. 146.5-147.5 ºC (chloroform); R_f_ 0.46 (1:3 ethyl acetate-heptanes); IR ν_max_/cm^-1^ 3143, 2844, 1617; ^1^H-NMRδ_H_ (400 MHz, CDCl_3_) 8.62 (dd, *J* = 3.9, 1.2 Hz, 1H), 8.47 (d, *J* = 2.6 Hz, 1H), 8.33 (s, 1H), 7.85 (dd, *J* = 4.9, 1.2 Hz, 1H), 7.45 (dd, *J* = 8.8, 2.6 Hz, 1H), 7.27 (dd, *J* = 4.9, 3.9 Hz, 1H), 6.91 (d, *J* = 8.8 Hz, 1H), 3.97 (s, 3H); ^13^C-NMR δ_C_ (100 MHz, CDCl_3_) 175.7, 164.9, 156.1, 151.9, 139.3 (2 signals), 136.8, 132.7, 132.1, 128.2, 125.2, 124.3, 113.5, 113.0, 55.9; *m/z* LRMS (ES+) 382.28 (100%, [M(^81^Br)+H]^+^), 380.25 (94, [M(^79^Br)+H]^+^), 316.28 (48), 314.27 (45), 298.26 (52), 296.25 (49).

*[4-(5-Bromo-2-methoxyphenyl)thiazol-2-yl](4-methoxyphenyl)methanone* (**3i**). Obtained on a 0.45 mmol scale as a yellow solid (134 mg, 74%). An analytical sample was recrystallized from chloroform to give pale yellow needles, m.p. 171.0-171.5 ºC (chloroform); R_f_ 0.39 (1:3 ethyl acetate-heptanes); IR ν_max_/cm^-1^ 3147, 2838, 1627, 1588; ^1^H-NMR δ_H_ (400 MHz, CDCl_3_) 8.65 (d, *J* = 9.1 Hz, 2H), 8.39 (d, *J* = 2.6 Hz, 1H), 8.30 (s, 1H), 7.45 (dd, *J* = 8.8, 2.6 Hz, 1H), 7.06 (d, *J* = 9.1 Hz, 2H), 6.92 (d, *J* = 8.8 Hz, 1H), 3.98 (s, 3H), 3.94 (s, 3H); ^13^C-NMR δ_C_ (100 MHz, CDCl_3_) 182.3, 166.6, 164.4, 156.2, 151.8, 133.9, 132.7, 132.0, 128.0, 125.0, 124.7, 114.0, 113.6, 113.2, 56.0, 55.7; *m/z* LRMS (ES+) 406.28 (100%, [M(^81^Br)+H]^+^), 404.26 (92, [M(^79^Br)+H]^+^).

#### 3.2.4. General procedure for demethylation with BBr_3_

The appropriate ketone (0.086 mmol) was suspended in dichloromethane (2 mL) and cooled to -78 ºC. Neat BBr_3_ (41 μL, 0.44 mmol) was added dropwise *via* microsyringe, whereupon the mixture turned black. The mixture was stirred at this temperature for 2 h and then allowed to gradually warm to r.t. over the course of 22 h. After cooling to -78 ºC, the reaction was quenched by careful addition of methanol (1 mL), saturated aqueous NaHCO_3_ (1 mL) and water (5 mL), followed by warming to ambient temperature. The mixture was extracted with ethyl acetate (3 × 10 mL), the combined organic layers dried (Na_2_SO_4_) and concentrated, and the resulting residue filtered through a short plug of silica gel (eluting with 1:3 ethyl acetate-heptanes) to give a crude product which was typically further purified by preparative HPLC and recrystallization from ethanol to yield the phenols **4a**, **4b** and **4d-i**.

*[4-(5-Bromo-2-hydroxyphenyl)thiazol-2-yl](phenyl)methanone* (**4a**). Obtained on a 0.086 mmol scale as pale yellow platelets (2.6 mg, 8%), m.p. 154.0-155.0 ºC (ethanol); R_f_ 0.23 (1:3 ethyl acetate-heptanes); IR ν_max_/cm^-1^ 3082, 1643, 1597, 1574; ^1^H-NMR δ_H_ (500 MHz, CDCl_3_) 10.93 (s, 1H), 8.22 (dd, *J* = 7.8, 1.3 Hz, 2H), 7.99 (s, 1H), 7.79 (d, *J* = 2.4 Hz, 1H), 7.69 (tt, *J* = 7.4, 1.3 Hz, 1H), 7.59 (ddt, *J* = 7.8, 7.4, 1.6 Hz, 2H), 7.38 (dd, *J* = 8.8, 2.4 Hz, 1H), 6.93 (d, *J* = 8.8 Hz, 1H); ^13^C-NMR δ_C_ (125 MHz, CDCl_3_) 184.1, 166.7, 155.1, 154.6, 135.1, 134.2, 133.7, 130.4, 129.0, 128.9, 120.2, 120.1, 118.6, 111.8; *m/z* LRMS (ES–) 360.25 (100%, [M(^81^Br)–H]^–^), 358.24 (88, [M(^79^Br)–H]^–^); HRMS (ES+) found 359.9693, C_16_H_11_^79^BrNO_2_S^+^ requires 359.9688.

*[4-(5-Chloro-2-hydroxyphenyl)thiazol-2-yl](phenyl)methanone* (**4b**). Obtained on a 0.58 mmol scale, from preparative HPLC of a 40 mg aliquat of a total of 123 mg crude product followed by recrystallization from ethanol, as pale yellow platelets (13 mg, 22% from aliquat), m.p. 139.0-143.5 ºC (ethanol); R_f_ 0.28 (1:3 ethyl acetate-heptanes); IR ν_max_/cm^-1^ 3082, 2925, 1728, 1640, 1596, 1576; ^1^H-NMR δ_H_ (400 MHz, CDCl_3_) 10.90 (s, 1H), 8.23 (dd, *J* = 7.7, 1.4 Hz, 2H), 7.99 (s, 1H), 7.71-7.56 (m, 4H), 7.25 (dd, *J* = 8.8, 2.6 Hz, 1H), 6.98 (d, *J* = 8.8 Hz, 1H); δ_C_ (100 MHz, CDCl_3_) 184.1, 166.7, 154.7, 154.6, 135.1, 134.2, 130.8, 130.4, 128.9, 126.1, 124.8, 120.2, 119.7, 117.9; *m/z* LRMS (ES–) 316.35 (38%, [M(^37^Cl)–H]^–^), 314.35 (100, [M(^35^C)l–H]^–^), 183.20 (40); HRMS (ES+) found 316.0189, C_16_H_11_^35^ClNO_2_S^+^ requires 316.0194.

*[4-(2,4-Dihydroxyphenyl)thiazol-2-yl](phenyl)methanone* (**4d**). Obtained on a 0.81 mmol scale (but the amount BBr_3_ used was 8 equivalents), from preparative HPLC of a 36 mg aliquat of a total of 150 mg crude product, as an orange, amorphous solid (29 mg, 50% from aliquat), m.p. 168.0 ºC (decomposition); R_f_ 0.11 (1:3 ethyl acetate-heptanes); IR ν_max_/cm^-1^ 3415, 3123, 2535, 2231, 1625, 1597; ^1^H-NMR δ_H_ (400 MHz, CD_3_OD) 8.33 (dd, *J* = 7.9, 1.3 Hz, 2H), 8.25 (s, 1H), 7.79 (d, *J* = 8.5 Hz, 1H), 7.70 (tt, *J* = 7.4, 1.3 Hz, 1H), 7.58 (tt, *J* = 7.7, 1.6 Hz, 2H), 6.41 (dd, *J* = 8.5, 2.3 Hz, 1H), 6.39 (d, *J* = 2.3 Hz, 1H); ^13^C-NMR δ_C_ (100 MHz, CD_3_OD) 185.5, 166.4, 160.6, 158.2, 157.0, 136.8, 134.7, 131.6, 130.2, 129.6, 120.9, 112.1, 108.8, 104.1; *m/z* LRMS (ES–) 296.38 (100%, [M–H]^–^), 165.20 (31); HRMS (ES+) found 298.0526, C_16_H_12_NO_3_S^+^ requires 298.0532.

*[4-(5-Bromo-2-hydroxyphenyl)thiazol-2-yl](4-methylphenyl)methanone* (**4e**). Obtained on a 0.31 mmol scale, after chromatography on silica gel (1:9 ethyl acetate-heptanes) followed by recrystallization from ethanol, as yellow needles (10 mg, 9%), m.p. 165.0-168.5 ºC (ethanol); R_f_ 0.46 (1:3 ethyl acetate-heptanes); IR ν_max_/cm^-1^ 3080, 1639, 1604; ^1^H-NMR δ_H_ (400 MHz, CDCl_3_) 11.0 (br s, 1H), 8.13 (d, *J* = 8.3 Hz, 2H), 7.96 (s, 1H), 7.78 (d, *J* = 2.4 Hz, 1H), 7.39-7.35 (m, 3H), 6.93 (d, *J* = 8.8 Hz, 1H), 2.47 (s, 3H); ^13^C-NMR δ_C_ (100 MHz, CDCl_3_) 183.5, 166.8, 154.9, 154.2, 145.3, 133.5, 132.3, 130.5, 129.6, 128.8, 120.0, 119.9, 118.5, 111.6, 21.9; *m/z* LRMS (ES–) 374.41 (83%, [M(^81^Br)–H]^–^), 372.39 (100, [M(^79^Br)–H]^–^); HRMS (ES+) found 373.9841, C_17_H_12_^79^BrNO_2_S^+^ requires 373.9845.

*[4-(5-Bromo-2-hydroxyphenyl)thiazol-2-yl](4-trifluoromethylphenyl)methanone* (**4f**). Obtained on a 0.23 mmol scale, after preparative HPLC followed by recrystallization from ethanol, as pale yellow platelets (15 mg, 15%), m.p. 138.0-138.5 ºC (ethanol); R_f_ 0.38 (1:3 ethyl acetate-heptanes); IR ν_max_/cm^-1^ 3099, 2359, 1658, 1576; ^1^H-NMR δ_H_ (400 MHz, CDCl_3_) 10.7 (br s, 1H), 8.32 (d, *J* = 8.7 Hz, 2H), 8.05 (s, 1H), 7.85 (d, *J* = 8.7 Hz, 2H), 7.79 (d, *J* = 2.4 Hz, 1H), 7.39 (dd, *J* = 8.8, 2.4 Hz, 1H), 6.93 (d, *J* = 8.8 Hz, 1H); ^13^C-NMR δ_C_ (100 MHz, CDCl_3_) 183.1, 165.6, 154.80, 154.77, 137.8, 135.2 (q, ^2^*J*_CF_ = 35.8 Hz), 133.8, 130.6, 128.9, 125.8 (q, ^3^*J*_CF_ = 3.7 Hz), 123.5 (q, ^1^*J*_CF_ = 272.9 Hz), 120.9, 120.0, 118.2; *m/z* LRMS (ES–) 428.26 (100%, [M(^81^Br)–H]^–^), 426.29 (95, [M(^79^Br)–H]^–^); HRMS (ES+) found 427.9571, C_17_H_10_^79^BrF_3_NO_2_S^+^ requires 427.9562.

*[4-(5-Bromo-2-hydroxyphenyl)thiazol-2-yl](4-chlorophenyl)methanone* (**4g**). Obtained on a 0.27 mmol scale, after preparative HPLC followed by recrystallization from ethanol, as orange platelets (15 mg, 14%), m.p. 162.0-163.5 ºC (ethanol); R_f_ 0.46 (1:3 ethyl acetate-heptanes); IR ν_max_/cm^-1^ 3104, 1634, 1584; ^1^H-NMR δ_H_ (400 MHz, CDCl_3_) 8.18 (d, *J* = 8.8 Hz, 2H), 8.00 (1s, 1H), 7.77 (d, *J* = 2.4 Hz, 1H), 7.56 (d, *J* = 8.8 Hz, 2H), 7.38 (dd, *J* = 8.8, 2.4 Hz, 1H), 6.92 (d, *J* = 8.8 Hz, 1H); ^13^C-NMR δ_C_ (100 MHz, CDCl_3_) 182.7, 166.1, 154.8, 154.5, 140.8, 133.7, 133.2, 131.7, 129.2, 128.9, 120.5, 120.0, 118.3, 111.8; *m/z* LRMS (ES–) 396.26 (29%, [M(^37^Cl^81^Br)–H]^–^), 394.31 (100, [M(^37^Cl^79^Br)–H]^–^, [M(^35^Cl^81^Br)–H]^–^), 392.36 (84, [M(^35^Cl^79^Br)–H]^–^); HRMS (ES+) found 393.9285, C_16_H_10_^35^Cl^79^BrNO_2_S^+^ requires 393.9299.

*[4-(5-Bromo-2-hydroxyphenyl)thiazol-2-yl](thiophen-2-yl)methanone* (**4h**). Obtained on a 0.14 mmol scale, after chromatography on silica gel (1:9 ethyl acetate-heptanes) followed by recrystallization from ethanol, as yellow platelets (52 mg, 89%), m.p. 165.0-166.0 ºC (ethanol); R_f_ 0.44 (1:3 ethyl acetate-heptanes); IR ν_max_/cm^-1^ 3085, 1616, 1510, 1409; ^1^H-NMR δ_H_ (400 MHz, CDCl_3_) 10.8 (s, 1H), 8.34 (dd, *J* = 3.9, 1.2 Hz, 1H), 7.98 (1s, 1H), 7.86 (dd, *J* = 4.9, 1.2 Hz, 1H), 7.77 (d, *J* = 2.4 Hz, 1H), 7.40 (dd, *J* = 8.8, 2.4 Hz, 1H), 7.28 (dd, *J* = 4.9, 3.9 Hz, 1H), 6.97 (d, *J* = 8.8 Hz, 1H); ^13^C-NMR δ_C_ (100 MHz, CDCl_3_) 174.7, 166.6, 154.6, 154.3, 138.2, 136.7, 136.3, 133.6, 129.0, 128.6, 120.5, 119.9, 118.5, 111.8; *m/z* LRMS (ES–) 366.35 (100%, [M(^81^Br)–H]^–^), 364.27 (64, [M(^79^Br)–H]^–^); HRMS (ES+) found 365.9248, C_14_H_9_^79^BrNO_2_S_2_^+^ requires 365.9253.

*[4-(5-Bromo-2-hydroxyphenyl)thiazol-2-yl](4-methoxyphenyl)methanone* (**4i**). Obtained on a 0.12 mmol scale, after preparative HPLC followed by recrystallization from ethanol, as yellow-orange platelets (3 mg, 6%), m.p. 146.0-147.0 ºC (ethanol); R_f_ 0.33 (1:3 ethyl acetate-heptanes); IR ν_max_/cm^-1^ 3097, 2916, 1633, 1596, 1568; ^1^H-NMR δ_H_ (400 MHz, CDCl_3_) 8.29 (d, *J* = 9.0 Hz, 2H), 7.95 (s, 1H), 7.78 (d, *J* = 2.4 Hz, 1H), 7.38 (dd, *J* = 8.8, 2.4 Hz, 1H), 7.06 (d, *J* = 9.0 Hz, 2H), 6.94 (d, *J* = 4.0 Hz, 1H), 3.92 (s, 3H); ^13^C-NMR δ_C_ (100 MHz, CDCl_3_) 182.2, 167.5, 164.7, 155.0, 154.2, 133.6, 133.1, 129.0, 127.7, 120.0, 119.8, 118.7, 114.4, 111.8, 55.8; *m/z* LRMS (ES–) 390.34 (100%, [M(^81^Br)–H]^–^), 388.39 (64, [M(^79^Br)–H]^–^); HRMS (ES+) found 389.9805, C_17_H_13_^79^BrNO_3_S^+^ requires 389.9794.

#### 3.2.5. Demethylation with cerium(III) ammonium nitrate

*[4-(2,4-Dihydroxyphenyl)thiazol-2-yl](phenyl)methanone* (**4c**). Ketone **3c** (27 mg, 0.083 mmol) was suspended in a mixture of acetonitrile (2 mL) and water (1 mL). Cerium(III) ammonium nitrate (110 mg, 0.20 mmol) was added in one portion and the resulting mixture stirred at r.t. for 1 h, at which point LC/MS analysis showed complete consumption of starting material. The mixture was diluted with water (5 mL) and extracted with diethyl ether (3 × 20 mL), the combined organic layers washed with brine (5 mL), dried (Na_2_SO_4_) and concentrated. The residue was dissolved in a solution of Na_2_S_2_O_3_ (450 mg, 2.8 mmol) in a mixture of water (3 mL) and dioxane (3 mL) and the resulting solution stirred at r.t. for 16 h. The mixture was diluted with water (5 mL), extracted with ethyl acetate (3 × 10 mL), the combined organic layers dried (Na_2_SO_4_) and concentrated, and the resulting residue purified by chromatography on silica gel (eluting with 1:3 ethyl acetate-heptanes) and recrystallization from acetone-heptane to give the hydroquinone **4c** as pale yellow platelets (10 mg, 41%), m.p. 191.0-192.0 ºC (acetone-heptane); R_f_ 0.06 (1:3 ethyl acetate-heptanes); IR ν_max_/cm^-1^ 3415, 3130, 2923, 1734, 1636, 1595, 1575; ^1^H-NMR δ_H_ (400 MHz, acetone-*d*_6_) 9.66 (s, 1H), 8.56 (s, 1H), 8.22 (dd, *J* = 7.7, 1.3 Hz, 2H), 7.95 (s, 1H), 7.76 (tt, *J* = 7.4, 1.3 Hz, 1H), 7.65 (t, *J* = 7.7 Hz, 2H), 7.52 (d, *J* = 2.8 Hz, 1H), 6.86 (d, *J* = 8.7 Hz, 1H), 6.80 (dd, *J* = 8.7, 2.8 Hz, 1H); ^13^C-NMR δ_C_ (100 MHz, acetone-*d*_6_) 184.7, 166.6, 155.9, 151.3, 149.7, 136.4, 134.5, 131.4, 129.5, 123.3, 119.4, 118.7, 118.5, 114.5; *m/z* LRMS (ES–) 296.34 (100%, [M–H]^–^), 165.18(35); HRMS (ES+) found 298.0532, C_16_H_12_NO_3_S^+^ requires 298.0532.

#### 3.2.6. Demethylation with dodecanethiolate

*[4-(5-Bromo-2-hydroxyphenyl)thiazol-2-yl](4-hydroxyphenyl)methanone* (**4j**). Ketone **3i** (40 mg, 0.10 mmol) and NaOH (24 mg, 0.60 mmol) were weighed into a 2 mL Emrys microwave vial. The vial was capped and flushed with nitrogen, followed by the addition of *N*-methylpyrrolidone (1 mL) and dodecanethiol (72 μL, 0.30 mmol) *via* syringe. The reaction mixture was subjected to microwave irradiation at a fixed temperature of 130 ºC (variable power) for 15 min. After cooling to ambient temperature, the reaction mixture was acidified with 1M HCl (until pH » 1) and extracted with ethyl acetate (3 × 5 mL). The combined organic layers were washed with brine (5 mL), dried (Na_2_SO_4_) and concentrated under reduced pressure. The crude residue was purified by chromatography on silica gel (gradient 1:9 to 1:3 ethyl acetate-heptanes) and preparative HPLC to yield the phenol **4j** as a pale yellow, amorphous solid (17 mg, 45%), mp 195.0-198.5 ºC; R_f_ 0.16 (1:3 ethyl acetate-heptanes); IR ν_max_/cm^-1^ 3370, 3093, 1706, 1628, 1591, 1441; ^1^H-NMR δ_H _(400 MHz, acetone-*d*_6_) 10.64 (br s, 1H), 8.67 (s, 1H), 8.38 (d, *J* = 8.9 Hz, 2H), 8.20 (d, *J* = 2.5 Hz, 1H), 7.40 (dd, *J* = 8.7, 2.5 Hz, 1H), 7.07 (d, *J* = 8.9 Hz, 2H), 7.00 (d, *J* = 8.7 Hz, 1H); ^13^C-NMR δ_C_ (100 MHz, acetone-*d*_6_) 182.5, 167.9, 163.9, 155.6, 153.7, 134.2, 133.4, 131.2, 127.7, 123.7, 121.5, 120.0, 116.4, 112.1; *m/z* LRMS (ES–) 376.30 (100%, [M(^81^Br)–H]^–^), 374.35 (78, [M(^79^Br)–H]^–^); HRMS (EI+) found 374.9560, C_16_H_10_^79^BrNO_3_^+^ requires 374.9565. 

## 4. Conclusions

In conclusion, we have synthesized a focused library of ten [4-(2-hydroxyphenyl)thiazol-2-yl]methanones according to a four-step procedure starting from 4-bromothiazol-2-ylcarboxaldehyde. The Suzuki coupling, addition of aryl Grignards, and the oxidation generally proceeded in modest to excellent yields. The final demethylation proved to be sluggish, but still produced enough material for characterization and biological evaluation. The target compounds were isolated in 2.2-24.4% total yields. None of the compounds were active as putative T3S inhibitors in whole-cell bacterial assays. The target protein has not been determined for the salicylidene acylhydrazides and all assays are therefore cell-based. This means that [4-(2-hydroxyphenyl)thiazol-2-yl]methanones that actually interact with the same target as the salicylidene acylhydrazides still do not turn up as an active due to other factors such as lack of permeability or rapid metabolism. In addition it cannot be excluded that the salicylidene acylhydrazides target more than one protein critical for T3S function and optimization of binding to more than one target by replacement of the central scaffold is likely to be challenging. Even if binding to one target is maintained or even improved, binding to other targets might be lost with lack of activity as overall result. Another explanation is that the thiazole scaffold does not properly mimic biophysical properties and the bioactive conformation of the salicylidene acylhydrazides. The compounds in the focused library are all novel and little information is published on related structures. A SciFinder search on the general functionalized thiazole structure shown in Figure 1d (performed 02-07-2010) resulted in ten substances in a total of three patents and one publication [20,21,22,23]. In addition, replacement of the salicylidene acylhydrazide scaffold with a mimic is of importance since the compound class has delivered biologically active molecules in a number of human and microbial systems as exemplified by several recent publications [24,25,26,27,28,29,30,31,32,33,34,35].
